# Case of a Man Who Castrated Himself

**Published:** 1797

**Authors:** Widdows Golding

**Affiliations:** Surgeon at Wallingford, in Berkshire, and Member of the Corporation of Surgeons in London.


VI. *Case of a Man who castrated himself. By Mr.* Widdows Golding, *Surgeon at Wallingford. Communicated in a Letter to Mr.* Thompson Forster, *Surgeon of Guy's Hospital, in London; and by him to Dr.* Simmons.

AGREEABLY to your request, I now communicate to you the case of the patient who, in a fit of religious enthusiasm, made an incision into his scrotum, and removed both his testicles.

He is an unmarried man, twenty-five years of age, and by trade a bricklayer.

He made the incision, with a pen knife, transversely at the bottom of the scrotum, so that both the testicles came out through the wound. He then divided each spermatic chord about an inch from the body of the testicle. A profuse hæmorrhage succeeded, which he attempted to stop by the application of stinging nettles to the part; but this not answering his expectations, he procured a needle and thread, with which he sewed up the external opening, and and thought all would be well, and that no perſon would know what had happened.

His friends, however, found out what he had done; and, after about five hours had elapſed, I was applied to, and immediately went to his aſſiſtance.

I found the ſcrotum, perinæum, and left iliac region diſtended with extravaſated blood.

I divided the whole of the tumefied ſcrotum, and let out above a quart of coagulated blood.

With much difficulty, owing to the retraction of the two chords, I paſſed a ligature round each of the ſpermatic arteries; and after applying a poultice of bread and milk to the part, ſecured by a tight bandage, I directed the abdomen to be fomented, and a ſuitable regimen to be obſerved. He took an opiate at night, and the next morning a purgative medicine.

March 17, 1795, (the day after the caſtration) I found him complaining much of pain. He had made water four times in the night, but had had no evacuation by ſtool. His pulſe was at 120, and full. The penis was much leſſened, and the ſcrotum was contracted. The purgative medicine was repeated.

18th: He had had three ſtools, and voided a ſufa ſufficient quantity of urine. His pulſe was at 125, and he complained of pain in the lower part of the abdomen. I obſerved a conſiderable ſwelling of the left ſpermatic chord, which had come on ſince the day before.

19th. His pulſe was 100. The left ſpermatic chord was ſtill much enlarged.

20th. His pulſe was 100. The ſwelling of the chord was decreaſed.

23d. The pulſe was 95. The uſe of the poultice and fomentation was diſcontinued, and ſimple dreſſings were applied to the wound, which granulated daily.

24th. About four ounces of coagulated blood came away laſt night from the right ſide of the wound, which had probably lodged about the abdominal ring. He was free from pain.

27th. The ligature of the right ſpermatic artery was removed, and three days after I removed the other ligature.

From the 21ſt of March he had taken freely of the bark; but on the 3d of April his health was ſo well reſtored, that medicine of every kind was diſcontinued, and before the middle of April he returned to his uſual employment.

*Wallingford,*

*June* 13, 1795.

